# Effect of screen time and outdoor activities on myopia progression

**DOI:** 10.1371/journal.pone.0347118

**Published:** 2026-05-08

**Authors:** Youssef Zougheib, Nagib Salameh, Andre Slim, Zahi Wehbi, Alaa Bou Ghannam, Christiane Al-Haddad

**Affiliations:** Department of Ophthalmology, American University of Beirut Medical Center, Beirut, Lebanon; University of the Highlands and Islands, UNITED KINGDOM OF GREAT BRITAIN AND NORTHERN IRELAND

## Abstract

**Objective:**

To assess the impact of screen time and outdoor activities on myopia progression in Lebanese children and to compare age groups.

**Methods and analysis:**

This prospective study enrolled 100 myopic children aged 3–17 years who presented to the pediatric ophthalmology service at the American University of Beirut Medical Center from February 2023 to January 2025. Behavioral data were obtained using a questionnaire, and clinical data were collected through retrospective chart review dating back to January 2018. Myopia was defined as a spherical equivalent (SE) ≤ −0.50 diopters (D). Annual myopia progression was compared during and after the COVID-19 lockdown and correlated with screen time and outdoor activity.

**Results:**

The mean age was 13.2 ± 3.6 years, with a balanced sex distribution. Myopic progression was significantly higher during the COVID-19 lockdown, with the highest progression in 2020–2021 (0.65 ± 0.07 D/year) compared with 2022–2023 and 2023–2024 (both 0.29 ± 0.05 D/year; p < 0.001). Mean SE became more negative over time, reaching −3.43 ± 0.23 D in 2024 (overall p < 0.001). During lockdown, screen time increased significantly (p < 0.001), while outdoor activity decreased significantly (p < 0.001). Twenty-two percent had more than 8 hours of daily screen exposure. Outdoor activity varied: 38% spent 5–10 hours outdoors weekly and 20% exceeded 10 hours. Younger children preferred tablets (p < 0.001) and spent less time on screens (p < 0.001). Nevertheless, questionnaire-derived daily screen time, weekly outdoor time, and screen‑break variables were not statistically significantly associated with myopia progression during or after the lockdown periods in the overall cohort (all p > 0.05).

**Conclusions:**

Myopia progression rate was higher during the COVID-19 lockdown than in the post-lockdown period, with significantly higher progression rates in 2020–2021 compared with 2022–2023 and 2023–2024. Screen time increased and outdoor time decreased during lockdown, but were not statistically significantly associated with progression in the overall cohort. These findings add evidence from an underrepresented Middle Eastern population, supporting further longitudinal studies of modifiable environmental factors in myopia progression.

## Introduction

Myopia, or nearsightedness, is the most widespread refractive error, developing primarily during childhood and early adulthood [1]. Despite being often regarded as a benign disorder, the World Health Organization recognizes uncorrected or under-corrected myopia as a main cause of visual impairment, with far-reaching implications for academic performance, productivity, and overall quality of life [2]. A recent meta-analysis projected a 39.8% increase in global myopia prevalence by 2050, underscoring the emerging status of this condition as a global health burden [3].

Historically, myopia has been considered primarily genetic, with only minor environmental influences [4]. However, increasing evidence over the past few decades has shifted this perspective. Near-work activities (reading, writing, watching television, etc.) have been associated with myopia onset and progression, although the underlying biological mechanisms remain incompletely understood and are likely multifactorial, including peripheral defocus signals [5]. Strong associations between myopia and years of schooling have been consistently observed [6]. In addition, urbanization and increasing pressures on academic performance have decreased outdoor playtime and physical activity, both of which are believed to play a protective role against myopia progression [7].

The widespread adoption of digital devices such as smartphones, tablets, and computers has further compounded these risks. Recent societal and technological changes have facilitated prolonged near work and artificial light exposure, contributing to the global rise in myopia prevalence [8,9]. The COVID-19 pandemic further intensified these challenges, as school closures and lockdowns necessitated online learning, hence increasing screen exposure among children [10]. A recent systematic review of pandemic-related lifestyle changes revealed a significant myopic shift among children aged 6–17 [11], attributed to increased digital device use and reduced outdoor time [12]. A previous study at our institution found a higher mean myopic spherical equivalent (SE) in 2020 and 2021 compared to previous years [13]. In children 3–10 years of age, annual myopic progression tended to be highest for 2019–2020 and 2020–2021 compared to previous years [13].

Outdoor activities have been shown to protect against the onset of myopia by minimizing the risk associated with near work [14] and genetic predisposition [15]. Studies indicate that an additional 40 minutes of daily outdoor time can reduce the incidence of myopia [16], with greater benefits observed within 80 minutes [17], suggesting a dose-response relationship. The most consistently proposed protective mechanism is higher light exposure outdoors. In contrast, evidence that outdoor time slows myopia progression after onset is mixed, with reports ranging from minimal or no effect to modest reductions [18]. These findings highlight the differential role of lifestyle modifications, particularly near-work patterns and outdoor exposure, in managing myopia progression. Therefore, the aim of the current study was to report myopia progression over time in a Middle Eastern population that is underrepresented in the myopia literature and to correlate it with screen time and outdoor activities.

## Materials and methods

### Study population

This study was approved by the Institutional Review Board at the American University of Beirut Medical Center (AUBMC) (IRB ID: BIO-2022–0186) and adhered to the principles of the Declaration of Helsinki. Written informed consent was obtained from the parents or legal guardians of the participating children. Child and adolescent assent forms were also provided.

The study involved prospective participant enrollment with retrospective data collection. We enrolled 100 myopic children aged 3–17 years presenting to the pediatric ophthalmology service at the AUBMC from February 2023 to January 2025. Demographic and behavioral data were collected at enrollment using a short 4–5-minute questionnaire (S1 Appendix) on areas of living, screen time, and outdoor activities. Behavioral patterns (screen time, screen breaks, and outdoor activity) were assessed once at enrollment and referred to two periods: the COVID‑19 lockdown/online-learning period (2020−2021) and the post-lockdown period (2022−2024). Clinical refraction data were extracted retrospectively from charts dating back to January 2018 to compute annual myopia progression. Spherical equivalent was calculated as sphere plus half cylinder in diopters (D). Myopia was defined as a SE ≤ −0.50 D. Patients with conditions that could affect myopia progression, including prior intraocular surgery, glaucoma, congenital cataract, and retinal disorders, were excluded. Patients with fewer than two visits or visits only after the COVID-19 lockdown were also excluded. The typical follow-up interval was once to twice annually, with annual visits used for analysis. Collected data included clinical and demographic factors (age, sex, past surgical or medical history, medications, family history, visual acuity, refraction, and fundoscopy). Age strata (3–10, 10–14, 14–17 years) were defined a priori to reflect clinically meaningful developmental stages. Subgroup sizes were not prespecified or balanced as this was a clinic-based sample and respective analyses were considered secondary to assess potential effect of heterogeneity by age.

### Eye examination

All patients underwent comprehensive ophthalmic examination, including visual acuity, cycloplegic refraction, slit-lamp examination, manual retinoscopy, and fundoscopy. Visual acuity was tested using vision charts (Allen pictures and Early Treatment Diabetic Retinopathy charts). Cycloplegia was achieved using tropicamide 1% and cyclopentolate 1%, applied twice, 10 minutes apart. Refraction was measured under cycloplegia using manual retinoscopy (cycloplegic retinoscopy), which is the standard method used in our pediatric ophthalmology clinic and was applied consistently across visits. Confirmatory automated cyclorefractions were additionally performed in older children when feasible. Annual myopic progression rate (D/year) was calculated as the absolute change in SE between two consecutive visits divided by the time interval in years. Progression during the COVID-19 lockdown was calculated as the mean annual progression across 2020–2021, and progression after the lockdown as the mean annual progression across 2022–2024.

### Statistical analysis

Data were recorded and analyzed using SPSS software (IBM SPSS Statistics for Windows, version 25.0; IBM Corporation). Means and standard deviations were calculated for continuous variables. Differences in model-estimated mean spherical equivalent and mean annual progression across years and intervals were assessed using linear mixed-effects models to account for repeated measures and inter-eye correlation, with p-values reported for the fixed effect of year/interval. Categorical variables were analyzed using chi-square test. For paired categorical comparisons between the lockdown (2020–2021) and after the lockdown (2022–2024) periods in the same participants, the McNemar-Bowker test was used. Subgroup analyses were performed for different age groups. Linear regression was applied on the right-eye data to study associated factors with myopia progression. A p-value of ≤ 0.05 was considered statistically significant.

## Results

### Study population

This study included 100 children diagnosed with myopia. Table 1 summarizes the demographic characteristics of our cohort. The mean age of our participants was 13.2 ± 3.6 years. Twenty-two children were between 3–10 years, 30 were 10–14 years, and 48 were 14–17 years. The sex distribution of our cohort was balanced, with 50% female participants. The median number of visits per patient was 4 (range: 2–7), and the median follow-up time was 11.8 months (range: 3.5–57 months).

**Table 1:** Demographic Characteristics of Study Participants.

	All participants (n = 100)
**Age (years), mean ± SD**	13.2 ± 3.6
**Age group, n (%)**	
3-10 years	22 (22%)
10-14 years	30 (30%)
14-17 years	48 (48%)
**Female sex, n (%)**	50 (50%)
**Number of visits, median (range)**	4 (2-7)
**Follow-up time (months), median (range)**	11.8 (3.5-57)

### Outdoor exposure, study environment, and screen time habits

Table 2 describes our cohort’s outdoor exposure, study environment, and screen time habits. Subgroup sizes (3–10 years: n = 22; 10–14 years: n = 30; 14–17 years: n = 48) were unequal (p < 0.001), reflecting the distribution of children presenting to our clinic. Most participants (50%) lived in Beirut, and 75% lived in city apartment buildings. Most of our cohort (78%) reported studying using an artificial light source (room or desk light), and only 5% reported not using an electronic device to study. The three age groups were compared in terms of response distribution to the screen time-related questions. Among the three age groups, significantly fewer children aged 14–17 years spent time outdoors. Outdoor time was significantly different across groups: 27.1% of children 14–17 years old spent more than 10 hours outdoors per week, as compared to 9.1% and 16.7% of the 3–10- and 10–14-years age groups, respectively. Study device was also significantly different among groups, with older children mostly using a laptop/PC while those between 3–10 years preferred tablets.

**Table 2:** Outdoor Exposure, Study Environment, and Screen Time Habits.

	All participants (n = 100)	3-10 years (n = 22)	10-14 years (n = 30)	14-17 years (n = 48)	p-value*
**Residence area, n (%)**					0.40
Beirut	50 (50%)	11 (50%)	16 (53.3%)	23 (47.9%)	
Mount Lebanon	28 (28%)	5 (22.7%)	9 (30%)	14 (29.2%)	
Other	22 (22%)	6 (27.3%)	5 (16.7%)	11 (22.9%)	
**Type of residence, n (%)**					0.34
City apartment	75 (75%)	16 (72.7%)	23 (76.7%)	36 (75%)	
Rural house	13 (13%)	2 (9.1%)	3 (10%)	8 (16.7%)	
Other	12 (12%)	4 (18.2%)	4 (13.3%)	4 (8.3%)	
**Weekly time spent outdoors, n (%)**					0.01
< 5 hours	42 (42%)	10 (45.5%)	10 (33.3%)	22 (45.8%)	
5-10 hours	38 (38%)	10 (45.5%)	15 (50%)	13 (27.1%)	
> 10 hours	20 (20%)	2 (9.1%)	5 (16.7%)	13 (27.1%)	
**Study lighting, n (%)**					0.85
Room/desk light	78 (78%)	18 (81.8%)	24 (80%)	36 (75%)	
Sunlight	5 (5%)	1 (4.5%)	1 (3.3%)	3 (6.3%)	
Both	17 (17%)	3 (13.6%)	5 (16.7%)	9 (18.8%)	
**Study device, n (%)**					<0.001
Laptop/PC	40 (40%)	5 (22.7%)	16 (53.3%)	19 (39.6%)	
Tablet	31 (31%)	9 (40.9%)	7 (23.3%)	15 (31.3%)	
Smartphone	10 (10%)	0 (0%)	3 (10%)	7 (14.6%)	
Multiple	14 (14%)	4 (18.2%)	4 (13.3%)	6 (12.5%)	
None	5 (5%)	4 (18.2%)	0 (0%)	1 (2.1%)	
**Most used device, (%)**					<0.001
Laptop/PC	15 (15%)	3 (13.6%)	4 (13.3%)	8 (16.7%)	
Tablet	24 (24%)	13 (59.1%)	9 (30%)	2 (4.2%)	
Smartphone	60 (60%)	5 (22.7%)	17 (56.7%)	38 (79.2%)	
Television	1 (1%)	1 (4.5%)	0 (0%)	0 (0%)	
**Daily screen time, n (%)**					<0.001
< 2 hours	8 (8%)	4 (18.2%)	3 (10%)	1 (2.1%)	
2-4 hours	19 (19%)	9 (40.9%)	7 (23.3%)	3 (6.3%)	
4-6 hours	20 (20%)	5 (22.7%)	7 (23.3%)	8 (16.7%)	
6-8 hours	31 (31%)	4 (18.2%)	7 (23.3%)	20 (41.7%)	
> 8 hours	22 (22%)	0 (0%)	6 (20%)	16 (33.3%)	
**Daily television time, n (%)**					0.002
< 2 hours	79 (79%)	13 (59.1%)	26 (86.7%)	40 (83.3%)	
2-4 hours	20 (20%)	9 (40.9%)	4 (13.3%)	7 (14.6%)	
4-6 hours	1 (1%)	0 (0%)	0 (0%)	1 (2.1%)	
**Screen time breaks, yes, n (%)**	73 (73%)	19 (86.4%)	20 (66.7%)	34 (70.8%)	0.07
**Break duration, n (%)**					0.06
< 5 minutes	4 (4%)	1 (4.5%)	1 (3.3%)	2 (4.2%)	
5-10 minutes	26 (26%)	5 (22.7%)	6 (20%)	15 (31.3%)	
10-30 minutes	22 (22%)	6 (27.3%)	9 (30%)	7 (14.6%)	
> 30 minutes	21 (21%)	7 (31.8%)	4 (13.3%)	10 (20.8%)	
**Bedtime device use, yes, n (%)**	82 (82%)	10 (45.5%)	26 (86.7%)	46 (95.8%)	<0.001

* p-value reflects differences in distribution across the three age groups.

The most frequently used device for any purpose was the smartphone (60%). Daily screen time also differed significantly, being least for the younger children (only 18.2% of children aged 3–10 years had 6–8 hours of daily screen time). Watching television was more common in children 3–10 years of age. Bedtime device use was significantly higher in older children, where 95.8% of those between 14–17 years reported using a device at bedtime.

### COVID-19 lockdown

Table 3 summarizes the screen time habits and outdoor activities during the COVID-19 lockdown period compared to the post-lockdown period in the same 100 participants. The vast majority of our cohort (92%) had online classes during lockdown, with 67% reporting that 2021 was the year with most online lessons, followed by 2020 (32%). Daily screen time during lockdown was significantly higher overall, with 47% reporting more than 8 hours compared to 22% after the lockdown (p < 0.001). A similar percentage (74%) reported having scheduled screen time breaks. Break duration differed significantly between the lockdown and post-lockdown periods (p < 0.001). Weekly time spent outdoors decreased significantly during lockdown, with 81% spending less than 5 hours outdoors compared to 42% post-lockdown (p < 0.001).

**Table 3:** Screen Time Habits and Outdoor Activities During and After the COVID-19 Lockdown.

	During lockdown	Post-lockdown	p-value*
**Online lessons, yes, n (%)**	92 (92%)	–	–
**Year with most online lessons, n (%)**		–	–
2020	29 (32%)		
2021	62 (67%)		
2022	1 (1%)		
**Daily screen time, n (%)**			<0.001
< 2 hours	5 (5%)	8 (8%)	
2-4 hours	9 (9%)	19 (19%)	
4-6 hours	18 (18%)	20 (20%)	
6-8 hours	21 (21%)	31 (31%)	
> 8 hours	47 (47%)	22 (22%)	
**Screen time breaks, yes, n (%)**	74 (74%)	73 (73%)	0.88
**Break duration, n (%)**			<0.001
< 5 minutes	0 (0%)	4 (5.5%)	
5-10 minutes	20 (27%)	26 (35.6%)	
10-30 minutes	40 (54.1%)	22 (30.1%)	
> 30 minutes	14 (18.9%)	21 (28.8%)	
**Weekly time spent outdoors, n (%)**			<0.001
< 5 hours	81 (81%)	42 (42%)	
5-10 hours	17 (17%)	38 (38%)	
> 10 hours	2 (2%)	20 (20%)	
**Type of residence, n (%)**			<0.001
City apartment	68 (68%)	75 (75%)	
Rural house	22 (22%)	13 (13%)	
Other	10 (10%)	12 (12%)	

* p-value for the McNemar-Bowker test for paired categorical data, comparing response distributions during lockdown versus post-lockdown. Percentages indicate the distribution of responses within each time period for the same cohort.

### Myopic progression

Table 4 shows the mean SE by year and the mean SE progression for the study intervals.

**Table 4:** Mean Spherical Equivalent By Year and Annual Progression.

	2018 (n = 7)	2019 (n = 46)	2020 (n = 48)	2021 (n = 51)	2022 (n = 80)	2023 (n = 81)	2024 (n = 70)	p-value*
Myopic spherical equivalent (D), mean ± SEM	−1.19 ± 0.30	−1.43 ± 0.24	−1.93 ± 0.24	−2.41 ± 0.24	−2.80 ± 0.23	−3.16 ± 0.23	−3.43 ± 0.23	<0.001
	**–**	**2018-2019 (n = 5)**	**2019-2020** **(n = 34)**	**2020-2021** **(n = 27)**	**2021-2022** **(n = 43)**	**2022-2023** **(n = 68)**	**2023-2024** **(n = 57)**	p-value*
Annual myopia progression (D/year), mean ± SEM	–	0.31 ± 0.17	0.44 ± 0.07	0.65 ± 0.07	0.45 ± 0.06	0.29 ± 0.05	0.29 ± 0.05	<0.001

* p-values from linear mixed‑effects models. Abbreviations: D, diopters; SEM, standard error of the mean.

In terms of mean SE, there was a significant difference across the years 2018–2024 (overall p < 0.001), indicating a progressive shift toward more myopic refraction from the pre‑lockdown period through the lockdown and post‑lockdown periods. Pairwise post-hoc testing with Bonferroni correction revealed that the mean SE showed a progressive increase in myopia over time: all between‑year differences were statistically significant (all p < 0.05), except for the comparison between 2018 and 2019, which was not significant (p = 1.00). Mean annual myopic progression was also significantly different overall (overall p < 0.001). Pairwise post-hoc testing with Bonferroni correction showed that progression rate during the lockdown period (2020–2021) was significantly higher than that in the post-lockdown period (2022–2023 and 2023–2024, both p < 0.001). All other between‑interval comparisons were not statistically significant.

Associations with behavioral exposures were evaluated separately for the lockdown and after lockdown periods. Progression during and after the lockdown were each tested against questionnaire variables pertaining to the respective time interval, including daily screen time, weekly time spent outdoors, and screen time breaks. During the lockdown, progression was not statistically significantly associated with daily screen time (p = 1.00), weekly outdoor time (p = 1.00), or screen time breaks (p = 1.00). After the lockdown, daily screen time (p = 0.49), weekly outdoor time (p = 0.26), and screen time breaks (p = 0.17) likewise showed no statistically significant associations with progression. Consistent with these findings, linear regression models, performed both in the overall cohort and stratified by age group, showed no significant associations between mean annual myopic progression and behavioral/environmental factors, including type of residence, daily screen time, screen-time break duration, or weekly time spent outdoors, in either time period (all p > 0.05).

## Discussion

This study investigated screen time habits, outdoor exposure, and myopia progression during and after the COVID-19 lockdown in a cohort of 100 school-aged children visiting the pediatric ophthalmology service at the American University of Beirut Medical Center. Overall, our cohort had significantly more screen time during the COVID-19 lockdown, with 47% reporting more than 8 hours of total daily screen time under lockdown conditions, compared to 22% after lockdown. Outdoor exposure was also significantly less overall, with 81% having less than 5 hours of weekly outdoors time during lockdown. Mean myopic SE for the whole cohort increased significantly over the years, reaching −3.43 ± 0.23 D in 2024. Myopia progression was significantly higher during the lockdown interval (2020–2021) than during the post‑lockdown intervals (2022–2023 and 2023–2024). Questionnaire-derived daily screen time, weekly outdoor time, and screen‑break variables were not statistically significantly associated with myopia progression during or after the lockdown periods in the overall cohort (all p > 0.05).

Multiple studies have evaluated the effect of screen time on myopia progression, with heterogeneous findings. A systematic review and meta-analysis found mixed results (pooled OR=1.02, 95% CI:0.96–1.08), where some studies reported an association between screen time and myopia, while others did not [12]. Another systematic review found that the evidence in the literature is conflicting and does not support a significant correlation between screen time and myopia, with studies also reporting variable results [19]. Increased time spent on near-work activities, such as studying, digital device use, or watching television, has been associated with higher odds of myopia [8]. A more recent systematic review with dose-response meta-analysis found that one additional hour of screen time was associated with higher odds of myopia, with a significant increase between 1 and 4 hours of daily screen time and a more gradual rise beyond 4 hours [20]. This association was observed across all age categories, but was highest in younger children, with an OR of 1.42 in children 2–7 years old, 1.12 for children 8–18 years old, and 1.16 for children 19 or older. A study in Japan found that myopia progression was not significantly associated with near work, outdoor time, or screen time [21]. In Algeria, excessive digital screen use was associated with significant increases in myopia of 0.50–0.70 D over 1 year. The study also found significant associations between device type, screen time, and reduced outdoor activity with myopia progression [22]. In our cohort, although screen time and myopic progression increased substantially during lockdown, behavioral variables collected by questionnaire were not significantly associated with progression when analyzed across the overall sample, either during lockdown or after lockdown (all p > 0.05). This is consistent with the heterogeneous literature and highlights the complex, multifactorial nature of myopia progression, where self-reported exposures may not fully capture cumulative or combined environmental effects.

The protective effect of outdoor exposure on myopia progression has been documented. In one study, students with higher levels of outdoor activity had significantly more hyperopic mean SE (+0.54 D) than those with low levels (+0.32 D) [14]. Interventional studies in China and Taiwan also showed significantly lower incidence and progression of myopia in groups with more time spent outdoors [7,16,17]. In our analyses, weekly outdoor time was not significantly associated with myopia progression in the overall cohort during either the lockdown or post-lockdown periods. This lack of association may be partly related to limited variability in outdoor exposure during lockdown, with 81% reporting less than 5 hours per week, as well as to measurement limitations inherent to retrospective self-reporting.

Many studies examined the effect of the COVID-19 lockdown on myopia in children. A study in China found that each additional hour of digital device use during the lockdown period was associated with a significant odds ratio of 1.25 for developing myopic symptoms [9]. Although this study focused on risk rather than progression, it supported the plausibility of an exposure-outcome link between increased digital device use and myopic change during lockdown. A systematic review found a pooled estimate of 0.73 D/year increase in myopia during the lockdown, which was statistically significant, in addition to a 2- to 2.6-fold increase in the incidence of myopia compared to before the COVID-19 lockdown [11]. The progression estimate from that meta-analysis is directly comparable in direction to our observed higher progression during 2020−2021. Time spent outdoors was lower (0.4–1.0 vs 1.1–1.8 hours daily), and screen time was higher (2.4–6.9 vs 0.7–2.8 hours daily) [11]. In the United States, the change in mean myopic SE from 2020−2021 was 2.2 times that from 2019−2020 [23]. This myopic shift outcome is methodologically closer to our interval-based progression estimates and similarly supports accelerated progression during the lockdown period. Another recent large study from China found that the prevalence of myopia increased by 11.3%, and the mean SE decreased by 0.36 D during the COVID-19 lockdown [24]. Because prevalence/incidence studies address myopia onset and population burden rather than progression among already myopic children, they are not directly comparable to our annual progression estimates; they are cited here as complementary evidence of a population-level myopic shift occurring in parallel with lockdown-related behavioral changes. A study in Saudi Arabia found that myopia progression increased during the pandemic, where all eyes exhibited a myopic shift during lockdown compared to a mostly hyperopic shift prior to the lockdown [25]. Time spent on near work activities was significantly higher during lockdown, with 35.8% spending 2−4 hours and 50.9% spending 4−6 hours on such activities, compared to 11.9% and 0% prior to the lockdown [25]. Time spent outdoors was significantly lower during lockdown, with 69.8% spending less than 1 hour, compared to 42.6% before lockdown [25]. A previous study from our group found that mean myopic SE increased over time, with 2021 showing a significantly higher SE (−2.39 D) compared to 2016, 2017, 2018, and 2019; and 2020 having a significantly higher mean SE (−2.21 D) compared to 2016 and 2017 [13]. There was no statistically significant difference in mean annual progression of myopia across the years. The results of this current study were similar in that mean SE increased over the years, reaching −3.43 D in 2024, a finding that may be partially explained by myopic progression with age. In contrast to the previous study, we found that mean annual SE progression was significantly higher during lockdown (0.65 ± 0.07 D/year) compared with the post‑lockdown interval (0.29 ± 0.05 D/year). Taken together, our findings are consistent with pandemic-era studies that measured refractive change or progression rather than prevalence, supporting an acceleration of myopic shift during the confinement period in association with increased screen-based near work and reduced outdoor exposure.

The strengths of our study lie in the use of a survey to collect data on the screen time habits and outdoor exposure during and after the COVID-19 lockdown, combined with long-term retrospective clinical follow-up using consistent cycloplegic refraction. The limitations of our study include the subjective measures of screen time habits and outdoor activities in the survey, which may have introduced recall bias as many surveys were completed several years after the COVID-19 lockdown. To mitigate this, the questionnaire used predefined time categories and was kept short to facilitate consistent reporting; nevertheless, some degree of recall error is possible and should be considered when interpreting exposure-outcome associations. Several patients missed one or more annual follow-up visits, which led to different sample sizes when comparing across years. The study design did not allow determination of a causal relationship. Studies with objective measures are needed to further investigate the relationship between screen time habits, outdoor exposure and myopia progression. Finally, residual confounding is possible. Although age and sex were accounted for in the analyses, other factors that may influence progression, including baseline refraction severity, parental myopia, and use of myopia control treatments, were not consistently available for all participants across the retrospective period and could not be reliably adjusted for.

This study highlights significant changes in screen time habits, outdoor exposure, and myopia progression among school-aged children during the COVID-19 lockdown. The cohort exhibited a substantial increase in daily screen time and a marked reduction in outdoor activity during lockdown, coinciding with accelerated myopia progression, particularly in 2020–2021 compared with 2022–2023 and 2023–2024. These findings align with global trends observed during the pandemic. However, no direct correlation could be demonstrated statistically, possibly due to the limited sample size and the influence of unmeasured or residual confounding factors that were not fully captured in the analysis. The study provides valuable evidence from an under-represented Middle Eastern population and underscores the need for further research to investigate the causal relationship between environmental factors, such as prolonged screen use and limited outdoor activity, and myopia progression, while also accounting for additional population-level confounders.

## Supporting information

S1 Appendix Survey Questionnaire. (DOCX)
