# Supplementary material for: Effect of screen time and outdoor activities on myopia progression
Source: PLoS One. 2026 May 8;21(5):e0347118. doi: 10.1371/journal.pone.0347118 (PMC13155576; doi:10.1371/journal.pone.0347118)
Supplement: S1 Appendix — (DOCX) [file pone.0347118.s001.docx]

1. **Age:**
2. **Gender:**

- **Male**
- **Female**

1. **Area of residence in Lebanon (in Winter):**

- **Beirut**
- **Mount Lebanon**
- **Beqaa**
- **South**
- **North**
- **Other**

1. **Area of residence in Lebanon (in Summer):**

- **Beirut**
- **Mount Lebanon**
- **Beqaa**
- **South**
- **North**
- **Other**

1. **Does your child take online classes?**

- **Yes**
- **No**

1. **During which year he/she took online classes the most?**

- **2019**
- **2020**
- **2021**
- **2022**

1. **What type of device does your child use for classes?**

- **Personal computer/Laptop**
- **Tablet**
- **Smartphone**
- **Multiple devices**

1. **How much time does your child spend on screen per day?**

- **Less than 2 hours**
- **2 to 4 hours**
- **4 to 6 hours**
- **6 to 8 hours**
- **More than 8 hours**

1. **What type of device does your child use the most?**

- **Personal computer**
- **Laptop**
- **Tablet**
- **Smartphone**

1. **How much time does your child spend on TV per day?**

- **Less than 2 hours**
- **2 to 4 hours**
- **4 to 6 hours**
- **6 to 8 hours**
- **More than 8 hours**

1. **Does your child take breaks while on screen or online?**

- **Yes**
- **No**

1. **If yes, for how much time?**

- **Less than 5 minutes**
- **5-10 minutes**
- **10-30 minutes**
- **More than 30 minutes**

1. **Do you live in an apartment or in a separate house?**

- **Apartment in city**
- **Apartment in a rural area**
- **House in city**
- **House in a rural area**
- **Apartment and house in different areas**

1. **Does your child usually go out or stay indoors?**

- **Go out during free time**
- **Go out in the night**
- **Stay Home during free time**
- **Stay Home most of the time**

1. **How much time does your child spend on outdoor activities per week?**

- **Less than 5 hours**
- **5 to 10 hours**
- **More than 10 hours**

1. **What kind of outdoor activities your child prefers to do?**

- **Walking/ running**
- **Biking**
- **Sport activities**
- **Health Clubs/Gym**
- **Other: specify**

1. **Does your child use electronic devices before bedtime?**

- **Yes**
- **No**

1. **What type of light does your child usually use while studying or working?**

- **Desk lamp**
- **Room light**
- **Natural light (sunlight)**

**During COVID Lockdown:**

1. **How much time did your child spend on screen per day?**

- **Less than 2 hours**
- **2 to 4 hours**
- **4 to 6 hours**
- **6 to 8 hours**
- **More than 8 hours**

1. **Did your child take breaks while on screen or online?**

- **Yes**
- **No**

1. **If yes, for how much time?**

- **Less than 5 minutes**
- **5-10 minutes**
- **10-30 minutes**
- **More than 30 minutes**

1. **How much time did your child spend on outdoor activities per week?**

- **Less than 5 hours**
- **5 to 10 hours**
- **More than 10 hours**

1. **Where did you live?**

- **Apartment in city**
- **Apartment in a rural area**
- **House in city**
- **House in a rural area**
- **Between apartment and house in different areas**
